# Exciton Luminescence and Optical Properties of Nanocrystalline Cubic Y_2_O_3_ Films Prepared by Reactive Magnetron Sputtering

**DOI:** 10.3390/nano12152726

**Published:** 2022-08-08

**Authors:** Anatoly Zatsepin, Yulia Kuznetsova, Dmitry Zatsepin, Chi-Ho Wong, Wing-Cheung Law, Chak-Yin Tang, Nikolay Gavrilov

**Affiliations:** 1 Institute of Physics and Technology, Ural Federal University, 620075 Ekaterinburg, Russia; 2 Institute of Metal Physics, Ural Branch of Russian Academy of Sciences, 620108 Ekaterinburg, Russia; 3 Department of Industrial and Systems Engineering, The Hong Kong Polytechnic University, Hong Kong; 4 Research Institute for Advanced Manufacturing, The Hong Kong Polytechnic University, Hong Kong; 5 Institute of Electrophysics, Ural Branch of Russian Academy of Science, 620049 Ekaterinburg, Russia

**Keywords:** nanocrystalline Y_2_O_3_ films, magnetron sputtering, luminescence, excitons, optical absorption, interband transitions, refractive index, thermal activation barriers

## Abstract

This paper presents a comprehensive study of the energy structure, optical characteristics, and spectral-kinetic parameters of elementary excitations in a high-purity nanocrystalline cubic Y_2_O_3_ film synthesized by reactive magnetron sputtering. The optical transparency gaps for direct and indirect interband transitions were determined and discussed. The dispersion of the refractive index was established based on the analysis of interference effects. It was found that the refractive index of the Y_2_O_3_ film synthesized in this study is higher in order of magnitude than that of the films obtained with the help of other technologies. The intrinsic emission of Y_2_O_3_ film has been discussed and associated with the triplet–singlet radiative relaxation of self-trapped and bound excitons. We also studied the temperature behavior of the exciton luminescence of Y_2_O_3_ for the first time and determined thermal activation barriers. The optical energy and kinetic parameters of cubic Y_2_O_3_ films were analyzed in comparison with those of the monoclinic films of yttrium oxide. The main difference between the optical properties of cubic and monoclinic Y_2_O_3_ films was established, which allowed for a supposition of their application prospects.

## 1. Introduction

Thin film oxides play an important role in emerging technologies such as photonics; micro-, nano-, and opto-electronics; as well as alternative energy sources [1,2]. Rare-earth oxides represent a promising group of multifunctional materials since they exhibit significant optical properties which are useful for commercial employment: high dielectric constant (~12–16), optical transparency over a relatively wide spectral range, and low phonon energy (up to 600 cm^−1^) [3]. Additionally, rare-earth oxides can be successfully involved in the manufacturing of laser devices, optical fibers, light-emitting diodes, high-frequency diodes, etc. [4]. One of the actively studied materials in this category is yttrium oxide (Y_2_O_3_), which is considered as a suitable host matrix for ionic dopants (including rare-earth and transition-metal ions) in order to construct and develop solid-state optical devices [5,6,7].

Three structural Y_2_O_3_ polymorphs are currently known: cubic, monoclinic, and hexagonal, which are commonly referred to as C-, B-, and A-type structures, respectively [8]. Among these crystal structures, cubic Y_2_O_3_ is the most stable phase under normal conditions (room temperature and atmospheric pressure). The optical properties of a cubic Y_2_O_3_ host in various modifications (bulk crystal, transparent ceramic, nanoparticles) have recently been studied in sufficient detail (e.g., see Refs. [9,10,11,12]). It has been found for bulk crystalline Y_2_O_3_ that the intrinsic luminescence of such a host matrix is associated with excitons and optically active defects. Additional interesting results concerning the interactions between excitons and ionic dopants have been reported as well. The energy transfer from self-trapped excitons to rare-earth ions in Y_2_O_3_ nanoparticles was discovered, and the quenching model of intrinsic luminescence in Y_2_O_3_ was proposed in Refs. [12,13]. As for the film-based morphology of yttrium oxide, data concerning the energy gap and the refractive index of Y_2_O_3_ were reported in Refs. [14,15] for radio frequency magnetron sputtering synthesis technology, electron beam deposition [16], thermal evaporation [17], and atomic vapor deposition techniques [18]. At the same time, the systematic studies on the emission properties of Y_2_O_3_ films are almost non-existent. It is for this reason that this is still one of the most challenging problems in this area of research since the information about the complex properties of the Y_2_O_3_ host matrix is highly essential for the onward development of functional materials for commercial applications.

The purpose of the current study is to discover the optical properties of Y_2_O_3_ thin films synthesized by reactive magnetron sputtering. We aimed to accomplish the following research tasks: to obtain high-purity films with low defectiveness; to study their energy structure and fundamental optical characteristics, including the spectral-kinetic parameters of elementary excitations; to compare our Y_2_O_3_ data with similar films synthesized by other technologies; and finally, to establish the influence of structural factors on the optical, energy, and kinetic parameters of nanocrystalline Y_2_O_3_ films. The data obtained will provide the necessary groundwork for the onward development of thin-film Y_2_O_3_ materials.

## 2. Materials and Methods

Y_2_O_3_ film was deposited onto a silica glass substrate using the dc-pulsed mode (50 kHz, 10 μs) of the reactive magnetron sputtering technology. Before the deposition process, the silica glass substrate was cleaned in an acetone solvent using an ultrasonic bath for 20 min, followed by drying in air. A target with a 40 mm diameter and a 2 mm thickness was yielded by employing the cold pressing procedure on metallic yttrium powder at 30 MPa. The magnetron, the sputtered target, and the substrate were placed in a vacuum chamber pumped down to 6.6 × 10^−3^ Pa with the help of a turbomolecular pump. Sputtering was carried out at 30 W magnetron power for 8 h, in an argon–oxygen atmosphere with a total pressure of 0.4 Pa and an oxygen volume concentration of less than 30%. The temperature of the substrate was maintained at 400 ± 25 °C during the deposition procedure. After deposition, the sample was cooled down to room temperature in a vacuum chamber with a pressure of 10^–5^ Torr. The thickness of the Y_2_O_3_ film was estimated to be at ~800 nm; it was determined by employing the ball-abrasion method with the help of the Calotest device (CSM Instruments SA, Peseux, Switzerland).

**Figure 1:**
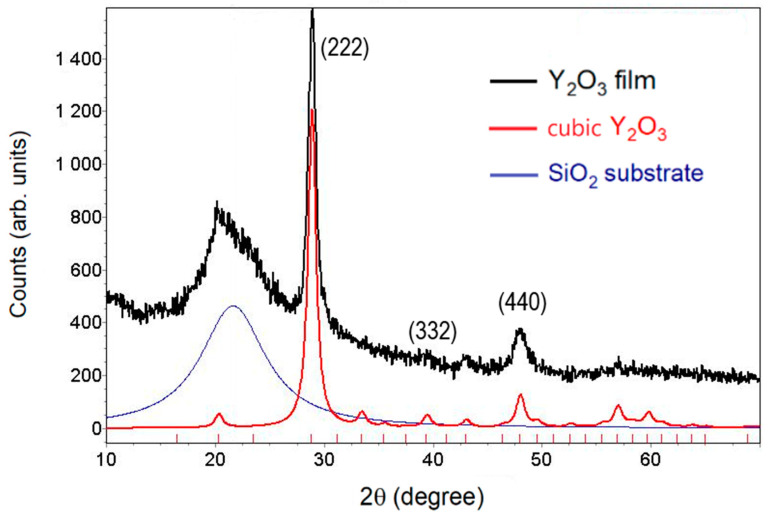
X-ray diffraction pattern of the Y_2_O_3_ film. Reflexes were identified in accordance with JSPDS No. 43-1036 Standard Card for the cubic phase of Y_2_O_3_. A wide diffused peak is formed due to the silica glass substrate.

The structural-phase analysis of the sample under study was performed by means of an X-ray diffraction technique using the XPertPro MPD diffractometer with Cu Kα = 1.5405 Å radiation. The processing of diffraction patterns was performed using the TOPAS 3 full-profile analysis program, taking into account the presence of a predominant orientation of crystallites (texture) in the material under the study. The diffraction pattern shown in Figure 1 can be identified based on one crystalline phase—the cubic Y_2_O_3_ (Ia-3 space group) with a lattice parameter of *a* = 10.73 Å. Plane (111), which is parallel to the surface, was found to be the preferred orientation. The wide diffused peak observed in the pattern is due to the silica glass substrate. No diffraction and reflection associated with the monoclinic modification of Y_2_O_3_ were found. The average size of 10 nm for the coherent scattering *D* was obtained from the Scherrer equation $D=\frac{k\lambda }{\beta \cos\theta }$, where λ is the wavelength of the X-ray, k is a constant equal to 0.89, θ denotes the Bragg angle, and β is the half-width of the line profile [19]. The average microstrain 〈ε〉 of the film was found to be 7.4 × 10^−3^ as $\langle \varepsilon \rangle =\frac{\beta \cot\theta }{4}$ [20]. In the Scherrer method used, the crystallite size and the magnitude of the microstrain were determined separately from the same (222) peak without considering the instrumental broadening. In this case, the obtained values of *D* and 〈ε〉 are the lower bound on the crystallite size and the upper bound on the microstrain, respectively. The theoretical density of the film was determined to be at 4.86 g/cm^3^ using the equation $\rho =\frac{ZM_{w}}{\mathrm{NV}}$ [21], where Z is the number of formula units per unit cell, $M_{w}$ is the molecular weight, V denotes the volume of unit cell, and N is Avogadro’s number.

**Figure 2:**
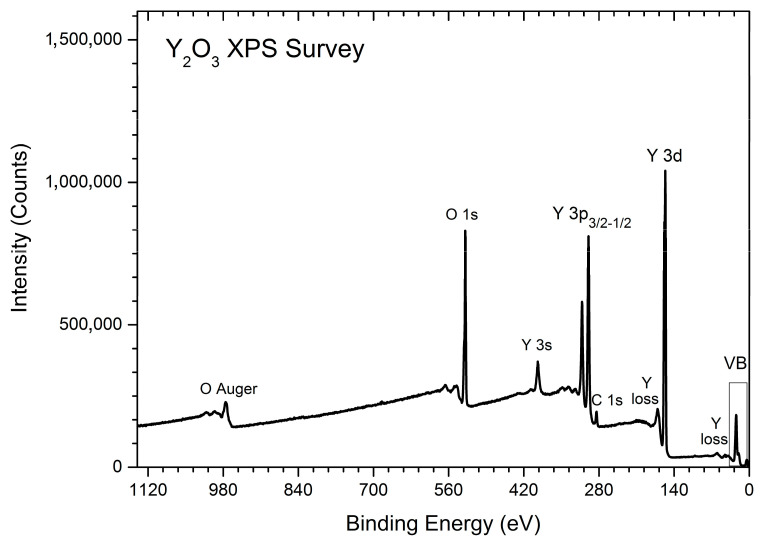
XPS Survey spectrum identification of the Y_2_O_3_ sample under study.

A chemical purity inspection of the sample under study was carried out using a ThermoScientific K-alpha Plus XPS spectrometer, which was equipped with a monochromatic micro-focused Al Kα X-ray source and had 0.05 at.% element sensitivity [22]. Operating pressure in an analytic chamber during a fast-wide scan (survey spectroscopy, 200 eV pass energy mode of a 180° hemispherical energy analyzer) was higher than 3.2 × 10^−6^ Pa. A dual-channel automatic charge compensator was applied to exclude the charging of our sample under XPS analysis because of the loss of photoelectrons. We performed pre-run-up procedures, including the standard degassing of the sample and analyzer, binding energy scale inspection, and re-calibration (when needed). In addition, we employed sputter-cleaned Au (4f_7/2_ band), Ag (3d_5/2_ band), and Cu (2p_3/2_ band) with built-in XPS Reference Standards according to the ISO 16.243 XPS International Standard and the XPS ASTM E2108-00 Standard. We used the built-in electronic database of ThermoScientific XPS spectrometer (ThermoFisher Scientific Waltham, Massachusetts, USA), the NIST XPS Standard Reference Database [23], and the Handbook of Monochromatic XPS Spectra: The Elements of Native Oxides [24] to precisely identify the survey spectrum structure (see Figure 2).

As can be seen in Figure 2, the most intensive peaks belong to yttrium and oxygen, which are the components of yttrium oxide. Carbon contamination (3.97 at.%) exists in the survey spectrum of the inspected Y_2_O_3_ sample, which is due to the well-known ability of Y_2_O_3_ to absorb CO and CO_2_ spices from the atmosphere (e.g., see Ref. [25]) and the general comments of ThermoScientific on the XPS study of yttrium oxide [26]. The measured O/Y ratio gives the value of 1.50, which is very close to the O/Y ratio = 1.51 of cubic Y_2_O_3_ [25]. The obtained value is dissimilar to the O/Y ratio = 1.46 of monoclinic Y_2_O_3_ reported in Ref. [27]. The XPS analysis of the survey spectrum confirms the formation of cubic high-purity Y_2_O_3_ film.

## 3. Results and Discussion

### 3.1. Interference and Refractive Index

**Figure 3:**
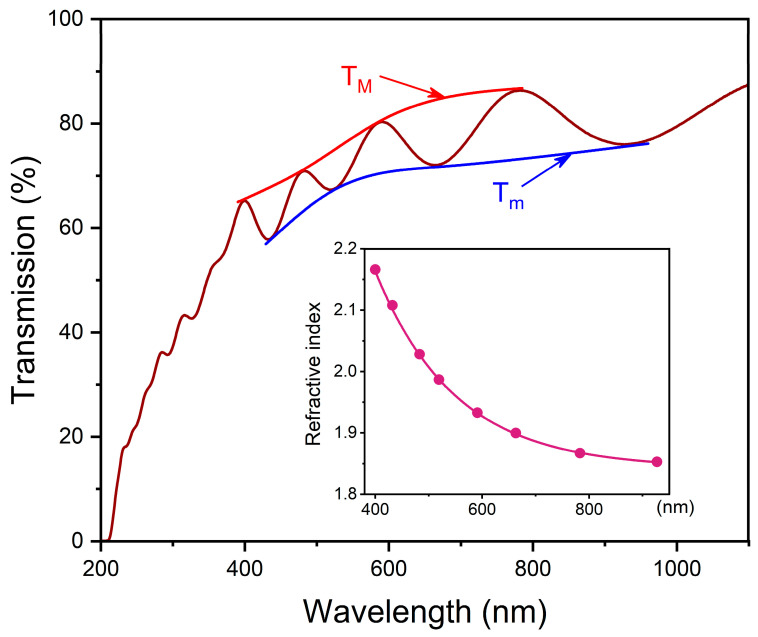
Optical transmission spectrum of Y_2_O_3_ film (brown line). Red and blue lines show the upper $T_{M}$ and lower $T_{m}$ envelopes of the spectrum, respectively. Inset shows dispersion of the refractive index obtained by Swanepoel’s method [28]. The error of the refractive index is ±0.005.

Figure 3 shows the optical transmission spectrum of Y_2_O_3_ film. The well-discernible interference extremes observed in the range of the optically transparent film indicate the high uniformity of the film and the clear-cut boundary between the film and the substrate. In order to determine the spectral dependence of the refractive index, we employed Swanepoel’s method [28] based on the analysis of interference effects. This method allowed for the construction of the upper $T_{M}$ and lower $T_{m}$ envelopes of the spectrum, which are shown in Figure 3 by the red and blue lines, respectively.

The values of the upper and lower “envelopes” are substituted in order to perform calculations of the refractive index n(λ):

(1) $$n(\lambda )=\sqrt{N(\lambda )+\sqrt{N{\left(\lambda \right)}^{2}-n_{s}^{2}}}$$

(2) $$N(\lambda )=2n_{s}\frac{T_{M}(\lambda )-T_{m}(\lambda )}{T_{M}(\lambda )\cdot T_{m}(\lambda )}+\frac{n_{s}^{2}+1}{2}$$

where N(λ) is the calculated intermediate value; $n_{s}$ denotes the refractive index of the substrate ($n_{s}=1.452-1.469$ for silica glass in the studied spectral range); $T_{M}(\lambda )$ and $T_{m}(\lambda )$ are the upper and lower “envelopes” of the optical transmission spectrum, respectively. The measured refractive index for the wavelengths corresponds to interference extrema and is represented by the circles in the inset of Figure 3. Spectral dependence n(λ) can be described using the simplified Cauchy equation (see violet line in the inset of Figure 3) [29]:

(3) $$n(\lambda )=n_{0}+\frac{A}{\lambda^{2}}$$

where $A=6.1\times 10^{4}\;\pm 0.1\times 10^{4}$ is a constant and $n_{0}=1.783\pm 0.005$ denotes the refractive index when λ→∞. The mean of refractive index dispersion is $D=n_{F}-n_{C}=0.117\pm 0.005$ where $n_{F}$ and $n_{C}$ are the refractive indexes at 486.1 nm and 656.3 nm, respectively.

In the targeted range of the spectrum, magnetron-sputtered cubic Y_2_O_3_ film produces a higher refractive index when compared with that for Y_2_O_3_ films synthesized with the use of other methods. For example, the refractive indices of cubic Y_2_O_3_ films synthesized by means of radio frequency magnetron sputtering [15], electron beam deposition [16], and thermal evaporation [17] in the range of 400–800 nm are n=1.95−1.91, n=1.93−1.89, and n=1.82−1.72, respectively. In addition, the Y_2_O_3_ film inspected in our study exhibits a relatively high dispersion of the refractive index, which is larger in order of magnitude than that reported in the literature for other Y_2_O_3_ films. It is known that refractive index is related to the porosity and packing density of a material [30]. One can estimate the porosity (*P*) and packing density ($P_{d}=1-P$) of the film using the known Lorentz–Lorenz expression [31]:

(4) $$\frac{n^{2}-1}{n^{2}+1}=\frac{n_{b}^{2}-1}{n_{b}^{2}+2}(1-P)$$

where n and $n_{b}$ denote the refractive index of the film and the bulk material, respectively. Assuming that the refractive index value of bulk yttria is $n_{b}=1.937$ at 590 nm [32], and accounting for experimental data obtained for the film, the porosity of the film was estimated to be P=0.003. The obtained value is smaller in order of magnitude than the value determined for films prepared by the electron beam deposition technique [16]. We believe that the increased refractive index value of our film is due to its high packing density.

Based on the spectral dependence of the refractive index, the thickness of Y_2_O_3_ film can be determined as follows:

(5) $$d=\frac{\lambda_{1}\lambda_{2}}{2(\lambda_{2}n_{1}-\lambda_{1}n_{2})}$$

where $n_{1}$ and $n_{2}$ are refractive indices at wavelengths $\lambda_{1}$ and $\lambda_{2}$, which correspond to the maxima (minima) of the neighboring interference observed in the transmission spectrum. Using several pairs of interference extrema, the average thickness of Y_2_O_3_ film is 812 ± 5 nm. The film thickness value obtained analytically is close to that declared during the synthesis stage (800 nm—ball-abrasion method). In the next stage of our research, we pay attention to the optical absorption of Y_2_O_3_ film.

### 3.2. Energy Gaps and Interband Transitions

**Figure 4:**
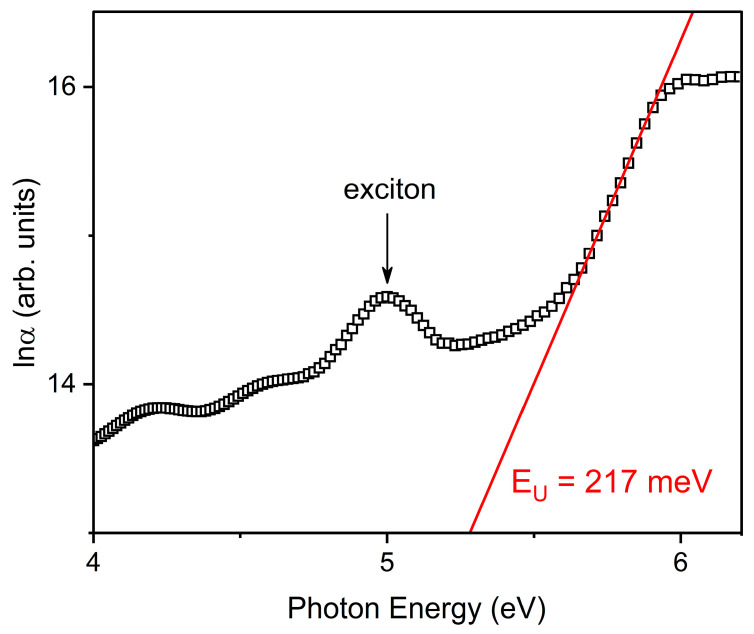
Spectral dependence of the absorption coefficient in the Urbach coordinates of the Y_2_O_3_ film. Red line shows an approximation of the linear range of lnα(hv) dependence. The Urbach energy $E_{U}$ was derived from Equation (6). The accuracy of $E_{U}$ calculation is ±2 meV.

Figure 4 shows the spectral dependence of the absorption coefficient in the tails of the region of the density of states, which was analyzed using the following expression for the Urbach rule [33]:

(6) $$\alpha (hv)=\alpha_{0}\cdot \exp\left(\frac{h\nu -E_{0}}{E_{U}}\right)$$

where $\alpha_{0}$ is a constant, $E_{0}$ denotes the coordinate of the crossing point of the so-called “crystal-like” Urbach rule, and $E_{U}$ is the Urbach energy denoting the measure of the overall structural disorder of the system under study (static and dynamic) [34]. The Urbach absorption edge is formed by optical transitions between the localized electronic states. The $E_{U}$ is a measure of the overall disorder of the material in our study. Based on the principle of equivalence and additivity of static (“frozen” phonons) and dynamic (“thermal” phonons) disorders, the Urbach energy reflects both the intrinsic irregularities of the lattice (i.e., defects, impurities, deviations from stoichiometry, etc.) and the thermal effects induced by the electron–phonon interaction [35,36].

The inverse slope of the linear range of lnα(hν) dependence refers to $E_{U}$ = 217 ± 2 meV (Figure 4). We have to note that the exact value of the Urbach energy was unknown for Y_2_O_3_. We compared the obtained $E_{U}$ value with that of other materials in the same family of rare-earth oxides–cubic gadolinium oxide films [37]. In our recent study, the Urbach energy of the Gd_2_O_3_ film was 483 ± 2 meV, and the intrinsic defects had significantly higher concentrations, which ensured a higher degree of structural disorder. As for the Y_2_O_3_ film, the Urbach energy was smaller, which indicates a relatively lower degree of total structural atomic disorder. In this case, a comparison of the Urbach energy for the Gd_2_O_3_ and Y_2_O_3_ films at room temperature gave a relatively good estimation of the total structural disorder, which is a combination of static and dynamic (phonon) disorders.

**Figure 5:**
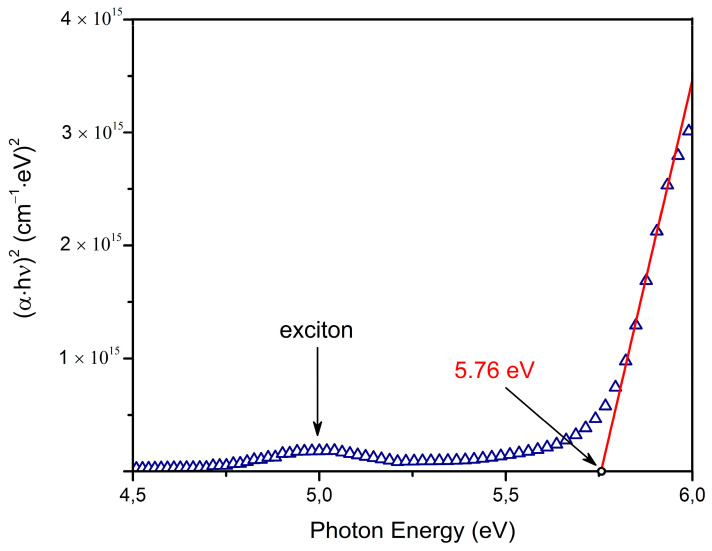
Optical absorption spectra of the Y_2_O_3_ film, shown in Tauc coordinates for direct allowed interband transitions. Arrows indicate the corresponding optical transparency gap with a ±0.01 eV accuracy. The band with its maximum at 5.0 eV is associated with exciton absorption.

The analysis of the fundamental absorption edge was performed with the help of the Tauc equation [38,39]:

(7) $${\left(\alpha \cdot hv\right)}^{n}=A\cdot (hv-E_{g})$$

where A is a constant; α denotes the absorption coefficient; hv is the photon energy; $E_{g}$ denotes the optical transparency gap, and n is the exponent which depends on the type of transitions. We studied direct allowed transitions at n = 2. The corresponding energy gap was determined by extrapolating the linear range of the ${\left(\alpha \cdot hv\right)}^{2}$ function to the intersection with the abscissa axis, as shown in Figure 5. On the one hand, the measured optical transparency gap is 5.76 eV, which is very close to the value of the optical transparency gap for the Y_2_O_3_ film synthesized by means of radio frequency magnetron sputtering ($E_{g}$ = 5.79 eV) [14]. On the other hand, the optical transparency gap of our sample is slightly larger than that for the Y_2_O_3_ film synthesized by employing atomic vapor deposition ($E_{g}$ = 5.60 eV) [18].

A band with its maximum at 5.0 eV was found in the absorption spectra (see Figure 4 and Figure 5). We believe that the origin of this band is associated with exciton absorption. It is known that for the bulk cubic Y_2_O_3_, a self-trapped exciton is formed by means of photon absorption and with an energy level of 6.0 eV [9]. As for Y_2_O_3_ nanoparticles with an average size of 10 nm, the exciton absorption band exhibits at 5.9 eV [40]. However, there are no data concerning the optical properties of excitons in Y_2_O_3_ thin films. The exciton absorption band of Y_2_O_3_ film is shifted to a low-energy region if compared with bulk materials and nanoparticles. The luminescent properties of excitons in Y_2_O_3_ film will later be studied in more detail.

### 3.3. Room-Temperature Exciton Emission

**Figure 6:**
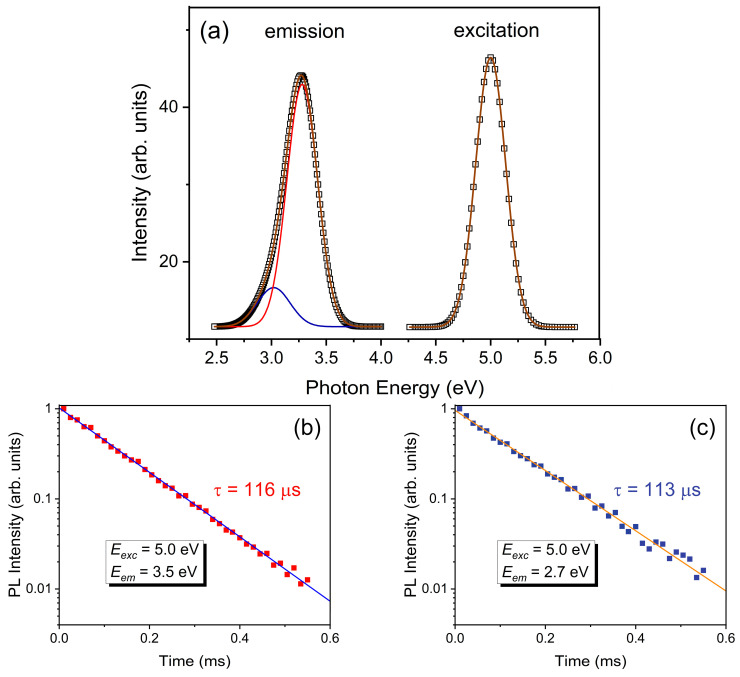
Room-temperature emission and excitation spectra of Y_2_O_3_ film. Red and blue lines show emission bands associated with radiative recombination of bound exciton and self-trapped exciton, respectively (**a**). Emission decay curves were measured at *E_em_* = 3.5 eV, *E_exc_* = 5.0 eV (**b**) and *E_em_* = 2.7 eV, *E_exc_* = 5.0 eV (**c**). The accuracy of the emission lifetime was ±3 μs.

Figure 6a shows the emission spectrum of the Y_2_O_3_ film under an excitation energy of 5.0 eV at room temperature. This emission spectrum is represented by the superposition of two bands whose maxima are located at 3.3 eV (FWHM = 0.3 eV) and 3.0 eV (FWHM = 0.3 eV). The excitation spectra of these two emission bands are similar to each other, and they produce one band with its maximum at 5.0 eV (FWHM = 0.3 eV). The locations of the excitation spectra were carefully calibrated. Otherwise, an overlapping of the spectra might have occurred.

For cubic Y_2_O_3_ in different morphologies (bulk crystal, transparent ceramic, nanoparticles), the luminescence at 3.4–3.5 eV is related to the radiative de-excitation of self-trapped excitons (*STE*) [9,11,12,41,42]. We believe that the dominant emission band located at 3.3 eV is also associated with the *STE* radiative recombination in Y_2_O_3_ film, and the spectral position of this band is shifted to the low-energy region when compared to bulk and low-dimensional cases. As for the second emission band, it is located at 3.0 eV, and the low-intensity luminescent band observed in the cubic Y_2_O_3_ is associated with the radiative recombination of the bounded exciton (*BE*), which was localized in the anion vacancy center of the *F*-type [11,42]. Thus, we believe that emission bands located at 3.3 eV and 3.0 eV are associated with *STE* and *BE*, respectively.

Figure 6b,c show the decay curves of exciton emission. The measurements were carried out by monitoring the emission wavelengths at the tails of the bands in order to avoid their overlap. The decay curves of both emission bands are single-exponential, and the emission lifetimes are 116 μs (E_em_ = 3.5 eV) and 113 μs (E_em_ = 2.7 eV). A large Stokes shift and slow luminescence decay kinetics indicate that the observed luminescence bands are associated with triplet–singlet radiative transitions. Triplet emission is usually characterized by a flare-up process because it overcomes the thermal activation barrier of the intersystem crossing (*ISC*) from a high-energy singlet state (*S_1_*) to a low-energy triplet state (*T_1_*) [43,44]. In the next section, we discuss our study of the temperature behavior of exciton-related emission.

### 3.4. Temperature Behavior of Exciton Emission: Activation Barriers

**Figure 7:**
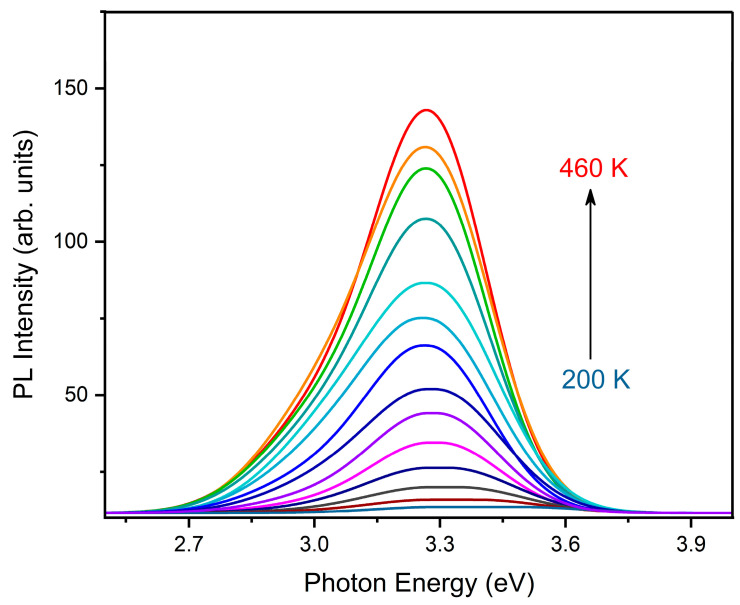
Temperature-dependent emission of Y_2_O_3_ film excited by 5.0 eV, with a temperature range of 200 K–460 K.

**Figure 8:**
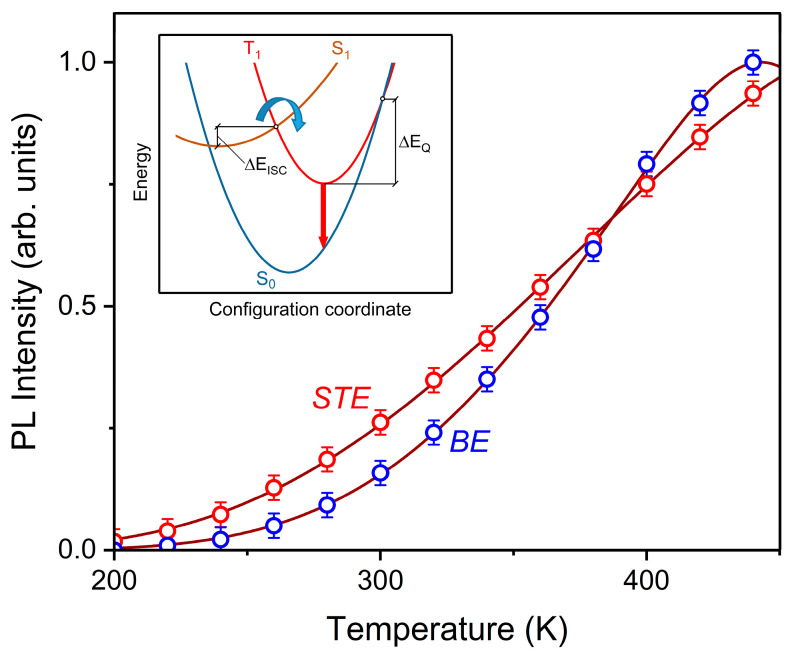
Normalized temperature dependences of emissions associated with self-trapped exciton (*STE*) and bound exciton (*BE*) in Y_2_O_3_ film. The circles are experimental data, whereas the lines are approximated by Equation (8). The inset shows a configuration coordinate diagram explaining the meaning of the thermal activation barriers $∆E_{ISC}$ and $∆E_{Q}$ of the intersystem crossing and emission quenching.

The temperature-dependent emission spectra of Y_2_O_3_ film excited by 5.0 eV are shown in Figure 7. It can be seen that emission appears at ~200 K, where its intensity increases with temperature growth. The maxima of bands have no significant changes in their energy locations and FWHMs. It can be seen that the temperature broadening of emission bands is absent, and this indicates a relatively weak exciton–phonon interaction. As a consequence, it produces a high-efficiency radiative relaxation. No new luminescence bands were found at low temperatures, which can be associated with singlet–singlet radiative transitions. This phenomenon probably occurs due to the small thermal activation barrier of the singlet–singlet non-radiative transition. Normalized temperature dependences of integrated intensities of luminescence bands whose maxima are located at 3.2 eV (*STE*) and 3.0 eV (*BE*) are shown in Figure 8. It can be noted that *BE*-related emission flares up at high temperatures and quenches at low temperatures. Hence, the thermal activation barriers of flare-up emission and luminescence quenching for *STE* and *BE* are certainly different.

In order to determine thermal activation barriers, we analyzed the temperature dependences of emission using the following analytical expression, which takes into account flare-up emission and luminescence quenching [43]:

(8) $$I(T)=\frac{I_{0}}{\left\{1+C_{1}\exp\left(\frac{∆E_{ISC}}{kT}\right)\right\}\cdot \left\{1+C_{2}\exp\left(\frac{-∆E_{Q}}{kT}\right)\right\}}$$

where $I_{0}$ is the maximum luminescence intensity if the quantum effects yield *ISC* and radiative recombination is equal to 1; $C_{1}$ and $C_{2}$ are kinetic factors of *ISC* and the triplet radiative transition, respectively; $∆E_{ISC}$ is the thermal activation barrier of *ISC*; $∆E_{Q}$ denotes the thermal activation barrier of emission quenching. Our experimental data are shown in Figure 8. The values of $∆E_{ISC}$ and $∆E_{Q}$ during *STE* emission are 131 meV and 482 meV, respectively. In contrast, the values of $∆E_{ISC}$ and $∆E_{Q}$ during *BE* emission are 189 meV and 412 meV, respectively (an accuracy of ±2 meV).

The *BE* emission is characterized by a larger flare-up barrier and a lower quench barrier when compared with the *STE* emission. The differences between these two thermal activation barriers are related to the non-identical adiabatic potential curves of *STE* and *BE*. A configuration coordinate diagram is shown in the inset of Figure 8. Symbols *S_0_*, *S_1_*_,_ and *T_1_* denote the singlet ground state, singlet excited state, and triplet excited state, respectively. A combination of factors, i.e., the emission energy, flare-up barrier, and quench barrier (*BE* versus *STE*), probably suggest that the triplet term of *BE* is more preferably shifted to the left side of the inset in Figure 8. The proposed model for the configuration curves assumes that the barrier of the singlet–singlet non-radiative transitions is very low since no new bands can be detected in the emission spectra even at liquid helium temperatures.

### 3.5. The Role of Structural Factors in the Formation of Optical, Energy, and Kinetic Parameters: Comparison of Cubic and Monoclinic Y_2_O_3_ Films

Comparative characteristics of the optical parameters of cubic and monoclinic Y_2_O_3_ films are shown in Table 1 and Table 2. The monoclinic Y_2_O_3_ film was synthesized using a similar technology, as described in Section 2; however, during the preparation of metallic yttrium powders, target sputtering sodium was used as an additive [45]. The purpose of adding sodium was to stabilize the monoclinic Y_2_O_3_ phase. The addition of alkali ions in rare-earth oxides is mainly used in order to create non-stoichiometry in the oxygen sublattice, which promotes the formation of the monoclinic Y_2_O_3_ phase [27,46]. The phase analysis performed confirms that the synthesized film is pure and has a monoclinic structure, and the average size of coherent scattering is 13 nm. We have highlighted the key features in Table 1 and Table 2.

Monoclinic Y_2_O_3_ film has a higher optical transparency gap than its cubic counterpart. The degree of atomic structural disorder is higher for monoclinic film and can be considered as evidence by the corresponding values of Urbach energy. This feature is likely due to the presence of defects in the monoclinic structure caused by sodium impurities embedded during fabrication. The refractive index of the cubic Y_2_O_3_ film is higher than that for monoclinic film because of the higher density of the cubic Y_2_O_3_ film.

The cubic Y_2_O_3_ film shows stronger emission energy for the exciton-related luminescent bands than the monoclinic polymorph of the Y_2_O_3_ film (see Table 2). Differences between the thermal activation barriers of *STE* and *BE* emission are similar to each other in terms of structural polymorphs: *BE* emission is characterized by a larger flare-up barrier and a lower quench barrier when compared with *STE*. As suggested above, this can be traced to the adiabatic potential curves of *STE* and *BE.* It is important to note that the thermal activation barriers of cubic Y_2_O_3_ in these two cases are relatively high when compared to those of its monoclinic counterpart.

In addition to exciton luminescence, the emission in the region of 3.4–3.8 eV is registered in the monoclinic Y_2_O_3_ film, which is associated with point defects in the anionic sublattice. Hence, the intensity of defect-related luminescence is comparable to the intensity of exciton-related luminescence. The formation of optically active defects in the monoclinic polymorph is due to the embedded sodium (impurity), which stabilizes the phase. In turn, no optically active intrinsic defects have been found in the high-purity cubic Y_2_O_3_ film, and luminescence occurs only due to the radiative relaxations of excitons.

Both cubic and monoclinic Y_2_O_3_ films have certain advantages and disadvantages. The film in a cubic structure has a lower optical transparency, but it shows a higher refractive index when compared with the monoclinic film. The exciton-related luminescence of cubic Y_2_O_3_ has a much higher quenching barrier, but it also flares up at higher temperatures as compared with the monoclinic Y_2_O_3_ film. In addition to exciton luminescence, the monoclinic Y_2_O_3_ film is characterized by defect-related emission, which is absent in the cubic polymorph. All these features should be considered when developing Y_2_O_3_-based thin-film materials for specific applications.

## 4. Conclusions

High-purity nanocrystalline Y_2_O_3_ film on a silica glass substrate was synthesized by employing the dc-pulsed mode of the reactive magnetron sputtering technique. This film has a cubic single-phase final structure, with a thickness of 800 nm and coherent scattering in the range of 10 nm. Comprehensive studies of the energy structure, optical characteristics, and spectral-kinetic parameters of the elementary excitations of Y_2_O_3_ film were performed.

Based on the interference effects of the optical transmission spectrum, the dispersion of the refractive index of Y_2_O_3_ film was determined. It was found that the refractive index (n = 2.165–1.859, λ=400–900 nm) of the studied film is higher than that of films synthesized by other methods. Parameters of the band energy structure of Y_2_O_3_ film were determined with the use of optical absorption spectroscopy. An analysis of the Urbach absorption edge showed a relatively low degree of structural atomic disorder for the cubic film in comparison to the film with a monoclinic structure. The optical transparency gap of the direct interband transitions was 5.76 eV.

Emissions at 3.3 eV and 3.0 eV in the Y_2_O_3_ film were associated with the triplet–singlet radiative de-excitation of self-trapped and bound excitons, respectively. It was found that the spectral positions of the exciton-related emission bands were shifted to the low-energy region when compared with the known bulk and low-dimensional Y_2_O_3_ morphologies. The temperature behavior of exciton luminescence in Y_2_O_3_ was studied for the first time. The thermal activation barriers of intersystem crossing and emission quenching were determined. It was established that the thermal activation barriers of both the flare-up and the quenching of exciton luminescence take on significantly higher values for cubic Y_2_O_3_ film when compared with its monoclinic counterpart.

The influence of structural types on the optical, energy, and kinetic parameters of Y_2_O_3_ nanocrystalline films was identified and discussed. The main features of the difference between the optical properties of cubic and monoclinic Y_2_O_3_ films determine the prospects for their practical use. The results obtained allow us to move a step forward to further develop optical materials that are based on Y_2_O_3_ thin-film polymorphs.

## Figures and Tables

**Table 1:** Fundamental optical parameters of cubic and monoclinic nanocrystalline Y_2_O_3_ films. $E_{g}$ is the optical transparency gap; $E_{U}$ denotes Urbach energy; n is the refractive index in the λ=400–900 nm range. Parameters of the monoclinic Y_2_O_3_ film were taken from Ref. [45].

Parameter	Cubic Y_2_O_3_	Monoclinic Y_2_O_3_
$E_{g}$, eV	5.76 ± 0.01	6.10 ± 0.01
$E_{U}$, meV	217 ± 2	515 ± 2
n (λ=400–900 nm)	2.165–1.859	1.621–1.532

**Table 2:** Spectral-kinetic parameters of elementary excitations in cubic and monoclinic nanocrystalline Y_2_O_3_ films. $E_{\mathrm{exc}}$ and $E_{\mathrm{em}}$ are the maxima of excitation and emission bands, which are associated with self-trapped exciton (*STE*) and bound exciton (*BE*); τ is the lifetime of an excited state; $∆E_{ISC}$ and $∆E_{Q}$ are the thermal activation barriers of intersystem crossing and emission quenching, respectively. Parameters of the monoclinic Y_2_O_3_ film were taken from Ref. [45].

Excitation	Parameter	Cubic Y_2_O_3_	Monoclinic Y_2_O_3_
STE	$E_{\mathrm{exc}}$, eV	5.00 ± 0.02	4.96 ± 0.02
	$E_{\mathrm{em}}$, eV	3.20 ± 0.02	3.13 ± 0.02
	τ, μs	116 ± 3	113 ± 3
	$∆E_{ISC}$, meV	131 ± 2	47 ± 2
	$∆E_{Q}$, meV	482 ± 2	193 ± 2
BE	$E_{\mathrm{exc}}$, eV	5.00 ± 0.02	4.96 ± 0.02
	$E_{\mathrm{em}}$, eV	3.00 ± 0.02	2.85 ± 0.02
	τ, μs	113 ± 3	129 ± 3
	$∆E_{ISC}$, meV	189 ± 2	80 ± 2
	$∆E_{Q}$, meV	412 ± 2	151 ± 2

## Data Availability

Exclude this statement.
